# Establishing a multidisciplinary initiative for interoperable electronic health record innovations at an academic medical center

**DOI:** 10.1093/jamiaopen/ooab041

**Published:** 2021-07-31

**Authors:** Kensaku Kawamoto, Polina V Kukhareva, Charlene Weir, Michael C Flynn, Claude J Nanjo, Douglas K Martin, Phillip B Warner, David E Shields, Salvador Rodriguez-Loya, Richard L Bradshaw, Ryan C Cornia, Thomas J Reese, Heidi S Kramer, Teresa Taft, Rebecca L Curran, Keaton L Morgan, Damian Borbolla, Maia Hightower, William J Turnbull, Michael B Strong, Wendy W Chapman, Travis Gregory, Carole H Stipelman, Julie H Shakib, Rachel Hess, Jonathan P Boltax, Joseph P Habboushe, Farrant Sakaguchi, Kyle M Turner, Scott P Narus, Shinji Tarumi, Wataru Takeuchi, Hideyuki Ban, David W Wetter, Cho Lam, Tanner J Caverly, Angela Fagerlin, Chuck Norlin, Daniel C Malone, Kimberly A Kaphingst, Wendy K Kohlmann, Benjamin S Brooke, Guilherme Del Fiol

**Affiliations:** 1 Department of Biomedical Informatics, University of Utah, Salt Lake City, Utah, USA; 2 University of Utah Health, Salt Lake City, Utah, USA; 3 Community Physicians Group, University of Utah, Salt Lake City, Utah, USA; 4 Department of Internal Medicine, University of Utah, Salt Lake City, Utah, USA; 5 Department of Family & Preventive Medicine, University of Utah, Salt Lake City, Utah, USA; 6 Department of Surgery, University of Utah, Salt Lake City, Utah, USA; 7 Department of Pediatrics, University of Utah, Salt Lake City, Utah, USA; 8 Department of Population Health Sciences, University of Utah, Salt Lake City, Utah, USA; 9 Division of Pulmonary Medicine, Department of Internal Medicine, University of Utah, Salt Lake City, Utah, USA; 10 MD Aware, LLC, New York, New York, USA; 11 Department of Emergency Medicine, New York University, New York, New York, USA; 12 Department of Pharmacotherapy, University of Utah College of Pharmacy, Salt Lake City, Utah, USA; 13 Intermountain Healthcare, Murray, Utah, USA; 14 Research & Development Group, Hitachi, Ltd, Tokyo, Japan; 15 Huntsman Cancer Institute, University of Utah, Salt Lake City, Utah, USA; 16 VA Center for Clinical Management Research, Ann Arbor, Michigan, USA; 17 Departments of Learning Health Sciences and Internal Medicine, University of Michigan, Ann Arbor, Michigan, USA; 18 VA Center for Informatics Decision Enhancement and Surveillance (IDEAS), Salt Lake City, Utah, USA; 19 Department of Communication, University of Utah, Salt Lake City, Utah, USA

**Keywords:** clinical decision support, interoperability standards, SMART on FHIR, FHIR, CDS Hooks, electronic health record

## Abstract

**Objective:**

To establish an enterprise initiative for improving health and health care through interoperable electronic health record (EHR) innovations.

**Materials and Methods:**

We developed a unifying mission and vision, established multidisciplinary governance, and formulated a strategic plan. Key elements of our strategy include establishing a world-class team; creating shared infrastructure to support individual innovations; developing and implementing innovations with high anticipated impact and a clear path to adoption; incorporating best practices such as the use of Fast Healthcare Interoperability Resources (FHIR) and related interoperability standards; and maximizing synergies across research and operations and with partner organizations.

**Results:**

University of Utah Health launched the ReImagine EHR initiative in 2016. Supportive infrastructure developed by the initiative include various FHIR-related tooling and a systematic evaluation framework. More than 10 EHR-integrated digital innovations have been implemented to support preventive care, shared decision-making, chronic disease management, and acute clinical care. Initial evaluations of these innovations have demonstrated positive impact on user satisfaction, provider efficiency, and compliance with evidence-based guidelines. Return on investment has included improvements in care; over $35 million in external grant funding; commercial opportunities; and increased ability to adapt to a changing healthcare landscape.

**Discussion:**

Key lessons learned include the value of investing in digital innovation initiatives leveraging FHIR; the importance of supportive infrastructure for accelerating innovation; and the critical role of user-centered design, implementation science, and evaluation.

**Conclusion:**

EHR-integrated digital innovation initiatives can be key assets for enhancing the EHR user experience, improving patient care, and reducing provider burnout.

## INTRODUCTION

Given the information-intensive nature of clinical medicine, it was long hoped that electronic health record (EHR) systems could help optimize health and health care.^1^ Indeed, it was anticipated that EHRs could help address key challenges such as patient deaths due to preventable medical errors,^2^ patients receiving as little as half of evidence-based recommended care,^3^^,^^4^ and medical costs increasing at unsustainable rates.^5^ To realize the promise of the EHR, many countries have invested heavily in EHR adoption.^6^ Following over $30 billion of government investment,^7^ the United States has achieved near-universal EHR adoption.^8^ Despite such heavy investments, EHRs have often failed to achieve their promise.^9^ While customization and training can improve the EHR user experience,^10^^,^^11^ physicians spend as much as 2–5 h on the EHR for every hour spent in direct patient care,^12^^,^^13^ and they can go through 4000 clicks per shift in the emergency department.^14^ In a recent study, 80% of physicians experienced physiological fatigue within the first 22 min of EHR use.^15^ Physicians often rate EHR usability as “unacceptable,”^16^^,^^17^ with low EHR usability contributing to stress and burnout.^9^^,^^18^ EHR adoption has not been associated with improved outpatient care quality in the United States.^19^^,^^20^ Clearly, the promise of EHRs remains unfulfilled.

**Figure 1.**
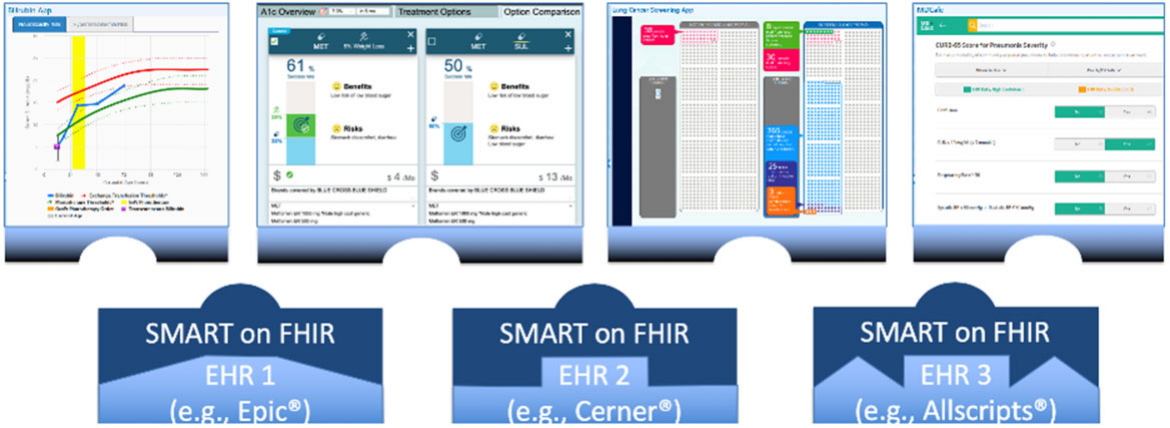
Architectural overview.

In seeking to realize the promise of EHRs, an exciting development is the evolution of EHRs from single-vendor products to vibrant platforms enhanced through third-party digital innovations (Figure 1). Just as smartphone users can augment their phones via apps, healthcare systems can increasingly augment their EHRs through digital innovations downloaded through EHR app stores.^21^^,^^22^ A key enabler for this innovation ecosystem is an interoperability standard known as SMART on FHIR (pronounced “smart on fire” and an acronym for Substitutable Medical Applications, Reusable Technologies on Fast Healthcare Interoperability Resources).^23^ SMART on FHIR allows digital innovations to be seamlessly integrated with the EHR’s native user interface through a single sign-on mechanism while reading and writing EHR data through standard application programming interfaces (APIs).

SMART on FHIR innovations can help meet clinician needs inadequately supported by the native EHR. Such needs can include support for high-level clinical reasoning, communicating and coordinating care, and complying with rules and regulations.^24^ Importantly, these innovations allow for rapid continuous improvement often not possible in the EHR itself. Because they are shareable via EHR app stores, duplication of effort can be reduced; and as they are substitutable with similar innovations, healthy competition can be promoted.

SMART on FHIR adoption is increasing, with use in over 500 hospitals^25^ and inclusion in federal regulations.^26^ Several healthcare systems have incorporated SMART on FHIR into their digital innovation strategy.^27–31^ Evidence is emerging that such digital innovations can improve clinical care.^27^^,^^32^^,^^33^ For example, a SMART on FHIR app for neonatal bilirubin management implemented at University of Utah Health (UUH) reduced the provider time required by two-thirds and was associated with significant improvements in guideline-compliant phototherapy.^27^

Knowledge sharing and coordination could enable more rapid progress in the adoption and impact of FHIR-based digital health innovations. However, beyond descriptions of individual innovations, there is a lack of literature on the larger initiatives producing these innovations and the lessons learned. To address this gap in the literature, we describe here the UUH ReImagine EHR initiative. UUH was an early innovator in the development, implementation, and evaluation of digital innovations leveraging SMART on FHIR and related interoperability standards. By describing the initiative and lessons learned, we hope to catalyze a more rapid transformation of health care through this promising approach to digital medicine.

## MATERIALS AND METHODS

### Setting

UUH is an academic health system with 5 hospitals and 12 community clinic centers. UUH has used the Epic^®^ EHR system-wide since 2014.

### Study design

Presented as a case study, we describe an enterprise initiative for EHR optimization at an academic health system leveraging SMART on FHIR and related interoperability standards. Key lessons learned are highlighted in the discussion. All human subjects research was approved by the Institutional Review Board (IRB).

### Manuscript structure

In the methods, we describe our motivation, facilitators, mission, vision, governance, structure, strategy, projects phases, and funding. In the results, we describe the developed infrastructure, individual innovations, and return on investment. In the discussion, we describe lessons learned.

### Motivation and facilitators

The primary motivation for launching ReImagine EHR was growing recognition that traditional EHR optimization methods were insufficient for fully meeting patient and provider needs. We also sought to better coordinate emerging efforts spanning both research and operations to maximize synergies. For example, research innovations must be translated into clinical practice to impact care. Similarly, difficult operational challenges can be addressed through research. We also aimed to increase our technical capacity to adapt to a rapidly changing healthcare landscape. Facilitators included the local availability of relevant experts as well as the increasing adoption of underlying standards by EHR systems including Epic^®^.^34^ A key catalyst for launching the initiative was the visionary leadership and resourcing support provided by health system leaders including the Chief Medical Information Officer (CMIO) and Chief Information Officer (CIO). This initial investment sparked substantial multidisciplinary research led by ReImagine EHR faculty in collaboration with investigators across the university.

### Mission, vision, governance, and structure

The mission of ReImagine EHR is to improve patient care and the provider experience. The focus is EHR-integrated digital innovations capable of widespread dissemination across diverse health systems and EHR platforms. Table 1 outlines our vision.

**Table 1.** ReImagine EHR vision

Imagine as a doctor	Imagine as a patient
*It is a joy to use the EHR* *The EHR is constantly saving you time* *It is easy to do the right thing, every time* *When you imagine how the EHR should work, it soon becomes how it does work*	*Your doctor is relaxed and has the time to address all your concerns* *You always receive excellent care based on the latest medical science* *Your medical care is individually tailored to who you are*

ReImagine EHR is a multi-stakeholder collaborative effort. The steering committee is co-chaired by the CMIO and CIO, and includes the Associate CMIO, the chair of the Department of Biomedical Informatics, the Director of Clinical Information Systems, the Director of Nursing Informatics, and the Chief Technology Officer. Key stakeholders are regularly consulted. ReImagine EHR is directed by the Associate CMIO.

The ReImagine EHR team includes 10 core members reporting to the director; other members contributing to many or most projects; and project-specific collaborators. The team includes clinical informaticists, physician leaders, cognitive psychologists, evaluation experts, and operational information technology leaders.

### Strategy and tactics

Core elements of the ReImagine EHR strategy include (1) establishing a world-class team; (2) creating supportive infrastructure; (3) focusing on the most impactful, sustainable, desirable, and feasible projects; (4) following best practices; and (5) maximizing synergies. Table 2 describes our strategy and associated tactics, rationale, and considerations. The core elements of our strategy were proposed by the director of the initiative (KK) and adopted by the steering committee at its first meeting. Since then, the strategy has been iteratively refined, with increased emphasis on evaluation and the pursuit of grant-funded research.

**Table 2.** Strategy, tactics, rationale, and associated considerations

Strategy	Tactics	Rationale	Considerations
Establish a world-class team	Recruit and retain experts in relevant domains including standards, clinical informatics, user-centered design, implementation, and evaluation	The underlying technology is cutting-edge and requires an expert workforce with technical as well as sociotechnical expertise	What expertise is needed for success? In what areas are we missing needed expertise?
	Support continuing training in needed skills (eg, training in EHR-specific API development)	The field is evolving rapidly; some technologies are EHR vendor-specific and require specialized training	Where is the field headed in terms of technological trends? Do we need additional training to meet current or expected needs?
Create enabling infrastructure to support individual innovations	Address common technical challenges through infrastructure tools	Tools that address common challenges can accelerate the development of individual innovations	What are the pain points in the development and implementation process? Is there a tool available to address the need, or do we need to create it?
	Create a systematic program for evaluating individual innovations across project phases	Evaluation is needed to ensure that innovations meet user needs, are adopted, and lead to desired outcomes. Standardization of evaluation steps saves resources and provides higher quality evaluation for individual projects	Are the tools being used? Do the tools meet users’ needs? How can we make them better? What impact are we having on users, patients and outcomes? What can we do to make future evaluations more efficient?
Focus on the most impactful, sustainable, desirable, and feasible individual innovations	Maximize return on investment with regard to patient care, finances, provider satisfaction, deployment scale, scientific impact, and/or research funding	To be sustainable, investments in EHR add-on apps must have a favorable return on investment	What is the anticipated impact in these areas? How many patients and/or providers could be positively impacted? Does the solution have commercialization potential? What is the cost of development, implementation, and sustainment? Will there be sufficient ongoing clinical value and/or potential for continuous external research funding to justify long-term institutional investment to sustain the innovation? Are additional resources (eg, grants) available? Will the project help foster the development of compelling new grant proposals?
	Focus on areas where desired functionality cannot be effectively and/or efficiently achieved through the native EHR, in particular cognitively complex decisions with limited EHR support	It is often easier and less expensive to configure the native EHR to support a specific task than to create an EHR add-on app. However, native EHR tools may be inadequate for meeting user needs related to cognitively complex decisions.	Does the EHR already do this sufficiently well? Has the EHR vendor committed to addressing this problem in an upcoming release? Is there a third-party product we should buy rather than building our own solution?
	Prioritize projects with a clear path to adoption	Lack of usage uptake is a common reason for an EHR add-on app to fail	Is there a committed clinical champion? Can the tool be integrated into routine clinical workflow? Can the tool be successfully deployed with minimal training?
Follow best practices for design, development, and implementation of individual innovations	Leverage interoperability standards such as FHIR, SMART, and CDS Hooks	While the use of standards can increase upfront development costs compared to using proprietary EHR configuration tools for which the workforce is predominantly trained, standards can reduce overall implementation costs and enhance dissemination potential	Is a relevant standards-based approach available for what we seek to accomplish? Is the marginal cost of standards support justified?
	Ensure security, privacy, and confidentiality	Securing patient privacy and confidentiality is paramount	What patient data are shared outside the institution? Are appropriate security controls in place? Are additional protections required, for example, to filter data shared with third parties through the FHIR protocol?
	Employ user-centered design and implementation	Innovations must meet user needs and be integrated with user workflows to succeed	What are the users’ needs in this area? What features would be most impactful for the user? What is the minimum viable product for early adopters? How will it best fit the users’ workflow?
Maximize synergies	Seek research synergies	An academic medical center has access to leading researchers; the field is a focal area for research	Are there researchers available with needed expertise? Are there research opportunities synergistic with what we are seeking to achieve?
	Consider partnerships	No single institution has the expertise and resources needed to fully optimize the EHR on its own	Does another group, either internal or external, possess relevant expertise or resources? Should we buy or license the tool? Does a partnership make sense?

### Project phases

Projects typically consist of 5 iterative and overlapping phases: Exploration; Design; Development; Implementation; and Sustainment and Dissemination. These phases are aligned with the Exploration, Preparation, Implementation, and Sustainment (EPIS) implementation science framework,^35^ with the EPIS Preparation phase encompassing our Design and Development phases.

*Exploration* consists of inception, prioritization, and resourcing. The ReImagine EHR Steering Committee prioritizes projects and allocates operational resources based on the principles outlined in Table 2. This exploration phase includes a “build vs. buy” analysis, in which we evaluate whether we have already purchased the needed capabilities, such as EHR modules that can be configured to meet user needs. When existing capabilities are insufficient, we evaluate third-party products and proceed to solution design and development only when the best course of action is to build the required functionality ourselves.

*Design* involves proposing a technology-facilitated solution to address user needs. ReImagine EHR uses an iterative, user-centered design process. Functional requirements are defined early based on an understanding of the nature of the clinical problem to be addressed. Both formal methods (eg, critical incident interviews, user workflow analyses, eye-tracking, card sorting, observation, heuristic evaluation, simulation studies with think-aloud, and/or focus groups) and informal methods (eg, stakeholder meetings, feedback from experts) may be used to assess user needs.^36–39^ Based on these needs, technical requirements are specified. User interface design is iteratively refined based on feedback from end users and socio-technical experts. The design is updated well into the implementation phase based on emerging user needs.

*Development* is conducted using the agile software development model. A typical “sprint” to develop additional functionality lasts 2 weeks, and all software updates undergo comprehensive testing prior to clinical deployment. Interoperability standards used include the Health Level Seven International (HL7) SMART standard for integrating the user interface, the HL7 US Core FHIR standard for data integration,^40^ and the HL7 Clinical Decision Support (CDS) Hooks standard for integrating point-of-care alerts and reminders.^41^ This phase is completed when testing is complete, governance approvals have been obtained, and an implementation plan has been developed.

*Implementation* involves operational use, refinement, and evaluation at initial clinical implementation sites. Typically, the application is first rolled out to pilot users to identify and address any major issues. A wider rollout then follows, with the system monitored and maintained to ensure proper functioning and updates being implemented as needed. The use, usability, efficiency, effectiveness, and impact of the system are formally or informally evaluated.

*The Sustainment and Dissemination* phase involves providing long-term support for innovations and extending reach and impact beyond the initial healthcare system. Most ReImagine EHR innovations are designed and developed with a goal of such dissemination. Dissemination may be conducted through EHR app stores or via institutional partnerships.

### Funding

ReImagine EHR is supported by both our health system and external funding. There are significant synergies between operations and research: innovations can be initially motivated by scientific questions and funded through research grants, then later evolve to support clinical operations. Likewise, innovations can be initially developed for clinical operations then lead to research projects, for example to further enhance a tool or to evaluate it through a multi-site trial. Regardless of the initial funding source, individual innovations must demonstrate sufficient clinical value to justify long-term operational support and maintenance. For projects supported by external funding, it is important to demonstrate clinical value during the project period if ongoing operational support is desired.

## RESULTS

ReImagine EHR was launched at UUH in January 2016. Below we describe enabling infrastructure developed through the initiative, 5 representative innovations, and return on investment to date.

### Challenges and developed infrastructure

We have encountered and resolved several challenges, many of which are technical in nature. Table 3 describes these challenges and the infrastructure developed to address these challenges. Additional approaches used and relevant considerations are also described. We are currently exploring potential approaches for making these infrastructure tools available outside the ReImagine EHR initiative.

**Table 3.** Challenges and associated infrastructure developed by ReImagine EHR

Challenge	Infrastructure addressing challenge	Additional approaches and considerations
EHR systems may not support desired FHIR data interfaces	FHIR Wrapper: tool that “wraps” an EHR’s native FHIR interface and provides support for additional desired FHIR interfaces, for example by making use of available non-FHIR data interfaces	Design applications so they can work with EHRs with differing levels of API support^42^ Help advance underlying interoperability standards and their adoption through leadership and service in organizations including HL7 (KK, GDF, CN) and the U.S. Health Information Technology Advisory Committee (KK)
EHR systems may support “standard” FHIR data interfaces differently	FHIR Wrapper: enables applications to interact with a consistent interface, with data requests and responses transformed as needed to accommodate divergent FHIR interface implementations specific to vendor products or product versions	Standards such as the US Core FHIR API^40^ still allow for substantial implementation flexibility, which can lead to divergent vendor implementations EHR vendors may offer only partial support for relevant standards
Sensitive data unnecessary for app functioning (eg, a patient’s HIV test results) may be transmitted to third-party apps by native EHR data interfaces due to a limitation of the current SMART on FHIR standard^43^	FHIR Wrapper: enables filtering out unnecessary data. For example, if a third-party app developer needs only the patient’s glucose levels but queries for all laboratory results or for glucose levels as well as HIV test results, the tool enables returning only the glucose levels	Host applications within the enterprise firewall Raise community awareness of this issue Advocate for addressing this issue through organizations including HL7 and the U.S. Health Information Technology Advisory Committee
Many tools require the definition of computable “value sets” containing a list of terminology codes that represent clinical concepts of interest	Terminology Suite: provides support for developing value sets in various domains, using available tools such as the National Library of Medicine’s Unified Medical Language System, RxNav, and Value Set Authority Center (VSAC)	Leverage VSAC value sets whenever appropriate. Modify these value sets when needed, and identify VSAC value set stewards that consistently provide high-quality value sets that require minimal or no modification upon detailed review (eg, organizations responsible for the development of national electronic clinical quality measures)
Significant effort is required to accurately map codes specific to a given EHR system or healthcare system to standard terminologies	EHR Mapping Tool: supports the mapping of local EHR data to standard codes expected by apps. For example, this tool can search through an EHR and identify all laboratory result types that contain the term “glucose.” The tool then displays relevant information such as frequency of use, example instances, units used, and context of use (eg, frequency of use within a basic metabolic panel versus a lumbar puncture)	Once a FHIR-based application has been developed, accurate terminology mapping is often the most time-consuming aspect of implementing the application in a given healthcare system. Consequently, a typical mapping approach may emphasize speed over accuracy (eg, identifying relevant codes solely by name or using a small number of examples to select and verify mappings). The goal of the EHR Mapping Tool is to enable rigorous terminology mapping comparable to an experienced analyst spending substantial time on each mapping, while significantly reducing the time required so as to make the approach practical for applying at scale
Applications used for patient care must be rigorously tested	Testing Suite: provides support for facilitating testing of standards-based EHR add-on apps, such as using FHIR payloads for testing and validating evaluation results delivered through the HL7 CDS Hooks standard. Due in part to the desire to facilitate such testing, the inferencing logic modules within our SMART on FHIR applications are often encapsulated within CDS Hooks services	Testing capabilities supported by our Testing Suite and associated tools include (1) the ability to develop and test against non-production EHR and FHIR server environments; (2) the ability to create de-identified FHIR data for testing, whether through user specification or de-identification of actual patient data; and (3) the ability to conduct regression testing using a large number of de-identified patient cases and their expected results
Rigorous evaluation is complex and expensive	A formal evaluation program including a systematic approach to evaluating digital health innovations across all project phases Recruitment of a Director of Evaluation for the initiative	Collectively, an evaluation team for interoperable EHR innovations should ideally possess masters or doctoral-level expertise in areas such as sociotechnical evaluation, data science, health services research, implementation science, statistics, health economics, and health outcomes evaluation

This supportive infrastructure has been iteratively developed to address challenges encountered in the pursuit of individual innovations, including those described in the section “Representative Innovations.” Some of these challenges impact most projects, including the need for value set specification, the need for EHR data mapping, and the need for rigorous testing and evaluation. Other challenges affect only a subset of projects, such as native EHR FHIR APIs lacking support for desired interfaces or transmitting unnecessary data to third-party applications hosted outside the enterprise firewall.

### Representative innovations

ReImagine EHR has engaged in the design, development, and implementation of over 10 applications to date. To illustrate how patient care and the provider experience across the care continuum can be improved through such innovations, 5 representative innovations are described below in terms of the problem addressed, the solution developed, the outcomes to date, and collaborating partners.

#### Neonatal bilirubin management app

*Problem:* Elevated bilirubin levels in newborns can cause brain damage. While a clinical guideline is available for neonatal bilirubin management,^44^ this management requires complex data synthesis and interpretation, and EHRs provide limited support for these tasks.

**Figure 2.**
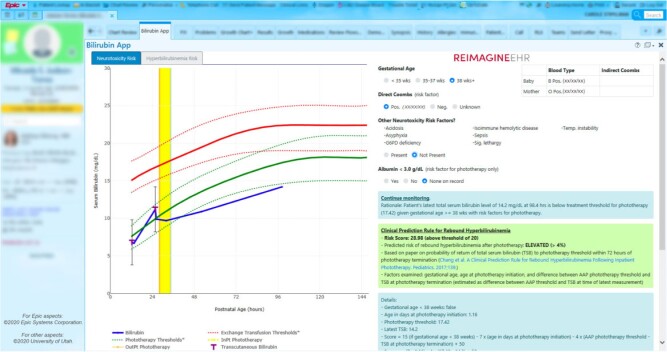
Bilirubin app.

*Solution:* The SMART on FHIR Bilirubin App (Figure 2) retrieves relevant information from across the chart, graphically displays these data, and provides patient-specific care recommendations according to the clinical guideline as well as risk predictions for rebound hyperbilirubinemia following phototherapy.

*Outcomes:* The Bilirubin App is used in the care of over 90% of applicable newborns at UUH.^27^ It was rated as having “best imaginable” usability by attending providers on the System Usability Scale, reduced the time needed for bilirubin management by 66%, and was associated with a significant increase in guideline-compliant phototherapy.^27^ The app has been recognized by several awards, including awards from the Department of Health and Human Services^45^ and the American Medical Informatics Association.^46^

*Partners:* UUH pediatricians, Intermountain Healthcare.

#### MDCalc connect

*Problem:* Clinical calculators are an essential tool for evidence-based care. While many clinical calculators are accessible through the Web, providers must manually search for the relevant input data (eg, age, gender, co-morbidities, vital signs, lab results) and enter the data into the calculator in a time-consuming and potentially error-prone process.

**Figure 3.**
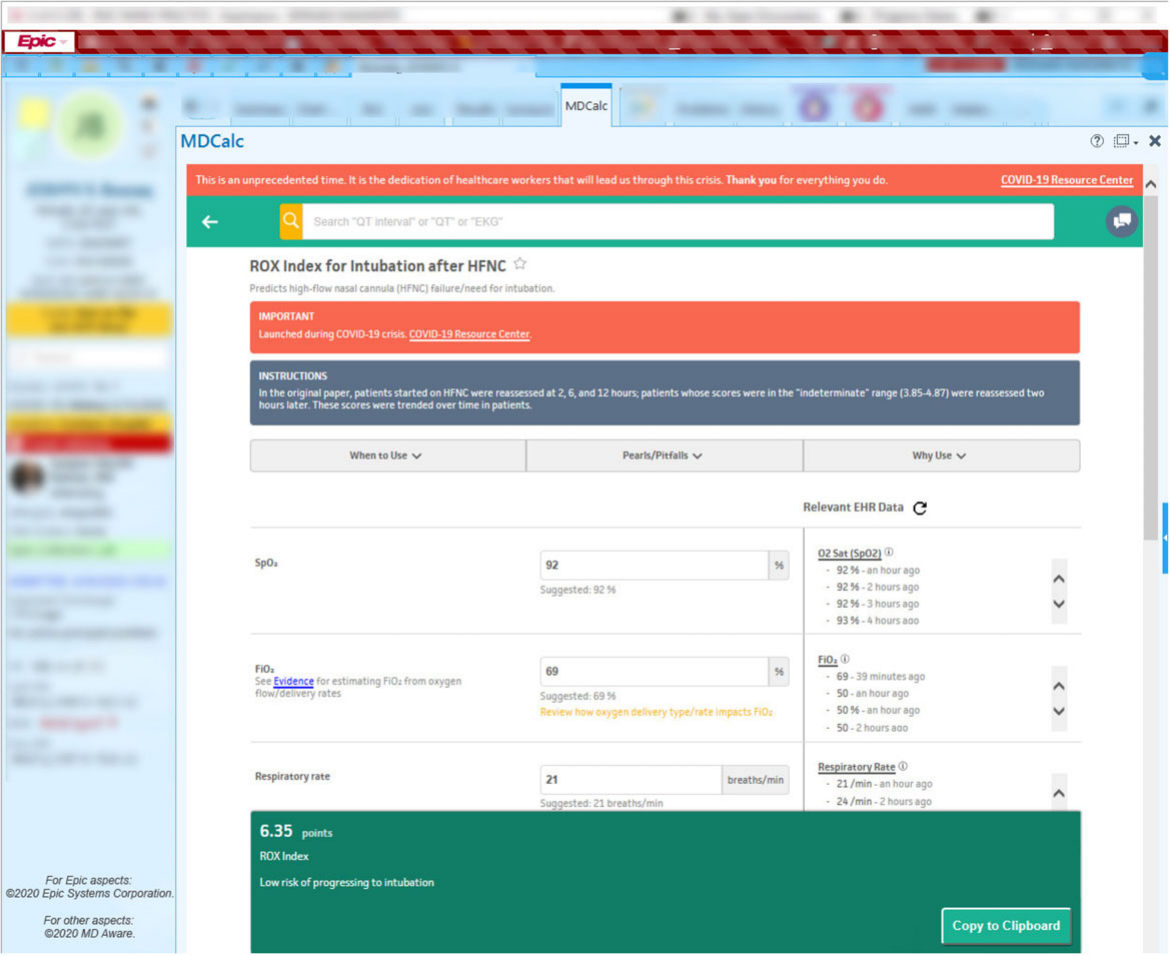
MDCalc connect.

*Solution:* MDCalc Connect, an EHR-integrated version of MDCalc, a leading clinical calculator platform historically available as Web and mobile apps (Figure 3). Calculator inputs are auto-filled with relevant EHR data in this SMART on FHIR app.

*Outcomes:* An initial evaluation of MDCalc Connect for the CHA_2_DS_2_-VASc calculator showed that EHR integration enabled the automatic identification of potential patient risk factors that were not otherwise noted by clinicians in more than half of patients.^47^

*Partners:* UUH providers and MD Aware, the makers of MDCalc. MDCalc covers over 200 conditions and is used by approximately two-thirds of US physicians.^48^

#### Diabetes pharmacotherapy outcome prediction app

*Problem:* Diabetes mellitus is a major source of mortality and morbidity, and pharmacotherapy is a core aspect of treatment. However, for most patients with diabetes, there is little evidence-based guidance on pharmacotherapy following initial treatment with metformin.^49^

**Figure 4.**
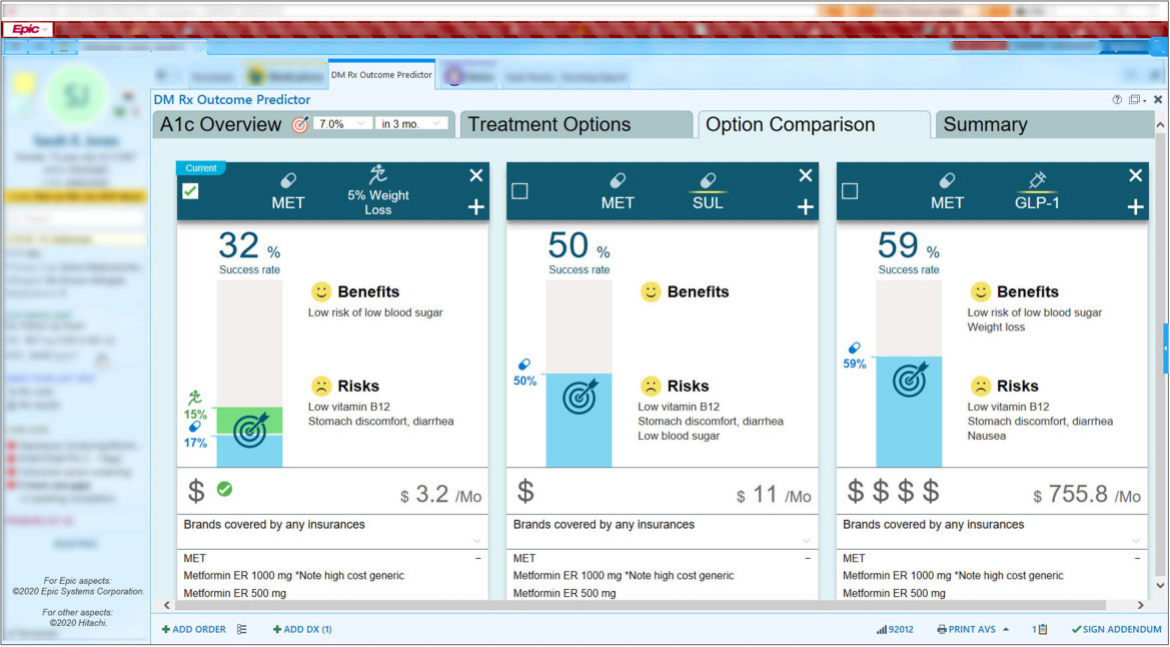
AI-facilitated diabetes decision support system.

*Solution:* Artificial intelligence (AI) was used to predict the medications most likely to be effective for individual patients to reach a patient-specific hemoglobin A1c goal. The application provides these predictions as well as information on relevant side effects and associated costs (Figure 4).^50^

*Outcomes:* A clinical trial is underway. The predictive models were found to have high predictive value in a validation data set, with an accuracy of 0.81 and an area under the curve of 0.88.^50^

*Partners:* Hitachi, Ltd. and the UUH Community Physicians Group.

#### Lung cancer screening shared decision-making app

*Problem:* Lung cancer is the leading cause of cancer deaths in the United States.^51^ Lung cancer screening with low-dose computed tomography scans could save more lives than breast cancer screening, and the US Preventive Services Task Force recommends screening to be considered for eligible patients with a heavy smoking history.^52–54^ However, screening rates among eligible patients in 10 US states was only 12.5% in 2017,^55^ due in part to the need for shared decision-making that considers patient-specific risks and benefits from screening.

**Figure 5.**
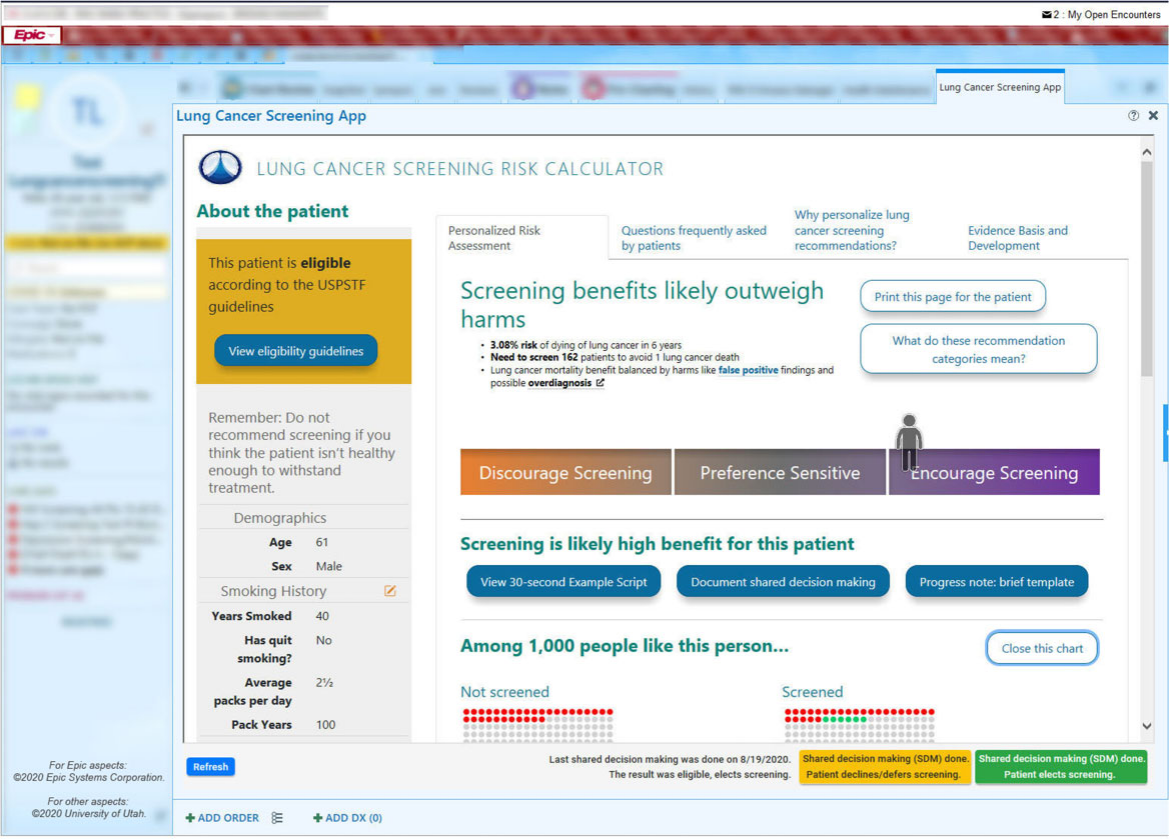
Lung cancer screening shared decision-making app.

*Solution:* To address this issue, ReImagine EHR developed an EHR-integrated version of Decision Precision, a lung cancer screening shared decision-making tool developed at the University of Michigan and the Department of Veterans Affairs (VA) (Figure 5).

*Outcomes:* An AHRQ-funded clinical trial at UUH is underway.

*Partners:* University of Michigan, Ann Arbor VA Center for Clinical Management Research, Salt Lake City VA Center for Informatics Decision Enhancement and Surveillance (IDEAS), UUH Community Physicians Group, UUH Department of Radiology.

#### Disease manager

*Problem:* Chronic diseases affect most adults, with many adults having multiple chronic conditions such as hypertension, diabetes, and chronic lung disease.^56^^,^^57^ Chronic diseases account for most morbidity and mortality in the United States, and significant clinical resources are required for managing these diseases.^58^ Despite their importance, EHRs provide limited cognitive support for chronic disease management, requiring clinicians to search for and organize disparate data throughout the EHR in order to obtain an adequate sense of the patient and the required clinical actions. As a result, patients oftentimes do not receive recommended care.^3^

**Figure 6.**
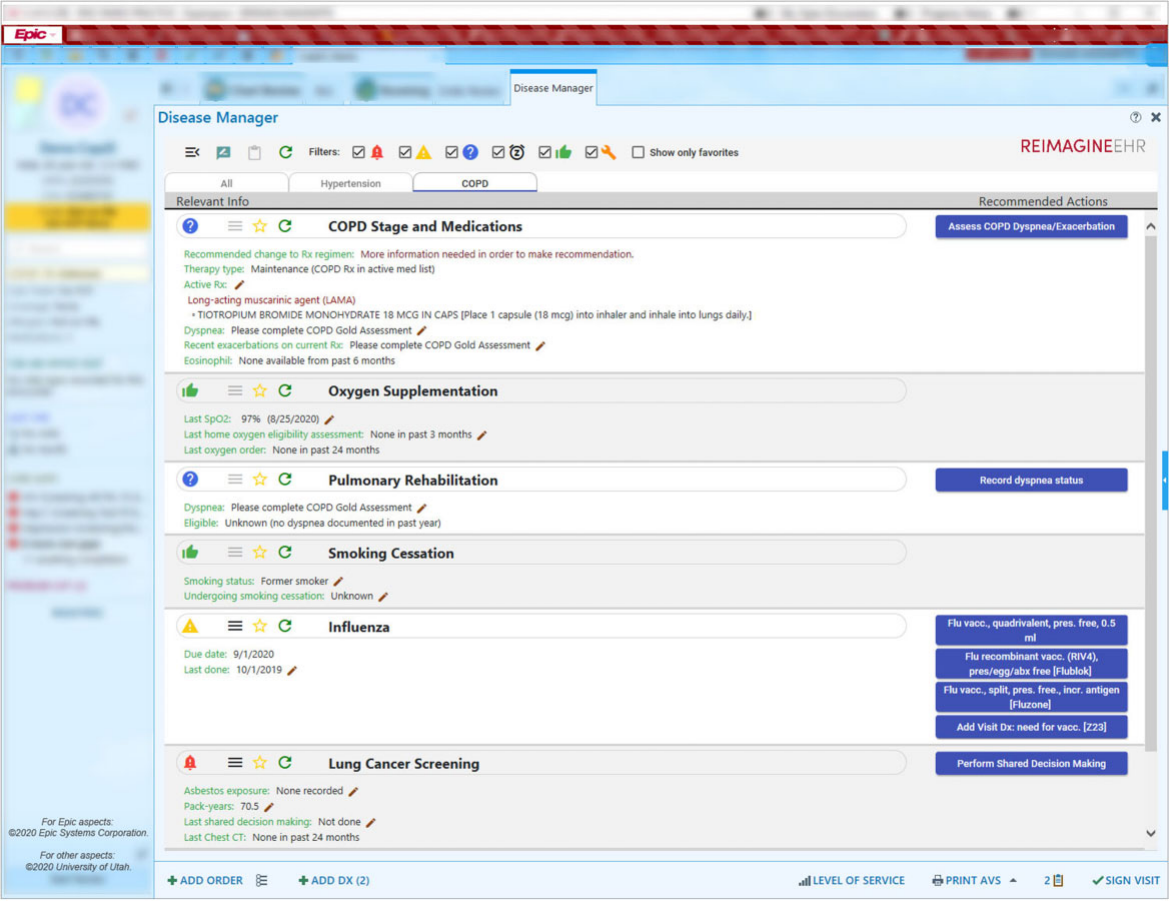
Disease manager.

*Solution:* A Disease Manager was developed to serve as a one-stop-shop in ambulatory care for chronic disease management and health maintenance. Initial modules supported include hypertension, diabetes, chronic obstructive pulmonary disease (COPD), and health maintenance (Figure 6). More than just an individual application, the Disease Manager is increasingly a foundational platform for other applications. For example, the Disease Manager incorporates both the Diabetes Pharmacotherapy Outcome Predictor App and the Lung Cancer Screening Shared Decision-Making App.

*Outcomes:* An experimental simulation study of this tool for COPD management found that compared to the EHR alone, the app was associated with improved completion of recommended care (81% vs. 48%, *P* < 0.001), reduced time spent per task, and reduced user frustration.^59^ A clinical trial is underway.

*Partners:* UUH primary care and specialist providers.

### Return on investment

Annual institutional investment in ReImagine EHR has been in the range of approximately $500 000 to $1 million, with almost all of the funds used to support the salaries of informaticists, software engineers, sociotechnical experts, and trainees. The return on this investment has included the positive impact of individual projects on patient care and the provider experience, grant funding, scientific outputs, and commercialization. To date, the initiative has helped to bring in over $35 million in external grants and contracts. Table 4 provides a summary of exemplar grants. ReImagine EHR also increases our health system’s capacity to adjust to change, whether it be a shift to value-based payment^60^ or a need to adapt to a pandemic. For example, in response to the COVID-19 pandemic, ReImagine EHR collaborated with its partners at MDCalc to develop a free SMART on FHIR application for COVID-19 management (Figure 3).^61^

**Table 4.** Representative external funding for ReImagine EHR

Funding source: project title (Principal Investigator Initials)	Project objectives
AHRQ R18HS026198: Scalable decision support and shared decision-making for lung cancer screening (KK)	To increase appropriate lung cancer computed tomography screening through the development and wide dissemination of EHR-integrated clinical decision support tools, including a SMART on FHIR shared decision-making app for lung cancer screening.
NCI U24CA204800: Scalable clinical decision support for individualized cancer risk management (GDF and KK)	To develop a standards-based population health management platform, with a focus on identifying, engaging, and managing patients who meet evidence-based criteria for genetic testing of familial breast and colorectal cancer. Standards used include FHIR and CDS Hooks.
NCI U01CA232826: Leveraging an electronic medical record infrastructure to identify primary care patients eligible for genetic testing for hereditary cancer and evaluate novel cancer genetics service delivery models (SSB and KAK)	To leverage the standards-based population health management platform described above to compare 2 approaches for delivering genetic counseling and testing for hereditary cancer to primary care patients: standard of care for genetic services or a self-directed approach assisted by an automated chatbot that provides education and explanation of results.
AHRQ U18HS027099: Enabling shared decision-making to reduce harm from drug interactions: an end to end demonstration (DCM)	To enable scalable shared decision-making dashboards that graphically communicate risks and decision options related to potential drug-drug interactions. Standards used include SMART on FHIR, CDS Hooks, and the Clinical Quality Language.
Hitachi: Clinical decision support system to optimize disease management (KK and CW)	To develop a standards-based, EHR-integrated diabetes management dashboard with predictive analytics about best treatment options and likely outcomes; and to conduct a clinical trial to evaluate the system’s impact. Standards used include SMART on FHIR and CDS Hooks.
CMS/Utah Department of Health (HITECH IAPD 182700537): Clinical health information exchange-based shared-care collaborative patient summary (CN and GDF)	To design, develop and implement a SMART on FHIR patient summary dashboard that integrates information from across healthcare systems through a statewide health information exchange to support the care of children with special healthcare needs.
ONC 90AX0013: Supporting closed-loop surgical referrals with a SMART on FHIR dashboard (BSB and GDF)	To implement a surgical referral SMART on FHIR dashboard that allows providers from different specialties to share a mental model of patient care and support surgical care transitions between the inpatient and outpatient settings.
NCATS 3UL1TR002538-03S4: Community-academic partnership to address COVID-19 among Utah community health centers (RH, DWW, and GDF)	To use multi-level CDS interventions (standardized symptom screening in the EHR, text messaging outreach to patients, and patient navigation) to increase the uptake of COVID-19 testing and immunization among underserved populations in community health centers throughout Utah.

AHRQ: Agency for Healthcare Research and Quality; CDS: clinical decision support; CMS: Centers for Medicare & Medicaid Services; FHIR: Fast Healthcare Interoperability Resources; NCATS: National Center for Advancing Translational Sciences; NCI: National Cancer Institute; SMART: Substitutable Medical Applications, Reusable Technologies.

## DISCUSSION

Digital innovations that extend native EHR capabilities have immense potential for improving health and health care. ReImagine EHR was among the first large-scale initiatives at an academic health system to embrace this approach to digital innovation. Key aspects of ReImagine EHR include a commitment to collaboration, a focus on delivering value, investment in human capital and infrastructure, the embrace of interoperability standards such as SMART on FHIR, and a high degree of synergy achieved across research and operations. As the healthcare community increasingly embraces digital innovation enabled by interoperability standards, we believe that our experiences can help guide future progress. Key lessons learned are described below.

### Investment in a digital innovation initiative leveraging SMART on FHIR can be valuable

By many metrics, ReImagine EHR has been a successful initiative: many innovative EHR add-on apps have been developed and implemented; long-term institutional funding has been sustained; substantial grant funding and corporate partnerships have been secured; and formal evaluations have demonstrated high user satisfaction and positive clinical impact.^27^ An initiative such as ReImagine EHR can serve as an important strategic asset for reducing cognitive load and addressing provider burnout. It can also help healthcare systems adapt to a rapidly changing landscape. We hope that the return on investment described in this manuscript can help fellow innovators at other organizations make the case for institutional investment in similar programs.

### High-value innovations can be implemented using only natively available EHR APIs

In some cases, valuable digital health innovations can be implemented across health systems using only the US Core FHIR APIs^40^ that are widely supported across EHR systems and required by federal regulations.^26^ For example, MDCalc Connect was designed to rely only on natively supported FHIR APIs to enable widespread dissemination.

### Non-native APIs can expand functionality but require more expertise and effort

There may be cases where APIs natively supported by the EHR are inadequate for meeting user needs. For example, for the bilirubin app, custom APIs were needed to support critical data such as the baby’s gestational age and phototherapy orders.^42^ Because substantial expertise and effort are required to develop these APIs, and because custom APIs make dissemination more difficult, the tradeoff between functionality and dissemination potential must be carefully considered.^42^

### Infrastructure tools can accelerate the pace of innovation

As described in Table 3, even when native EHR APIs meet all data needs, other technical challenges often arise. Infrastructure tools addressing such needs support the efficient design, development, and implementation of interoperable digital health innovations.

### User-centered design is critical to achieving high adoption and desired impact

Critical to achieving wide uptake and impact is a systematic and iterative approach to user-centered design. With the help of experts in cognitive psychology, we elicit user needs, design our solutions to meet those needs and associated workflows, and make iterative enhancements based on user feedback. Key user needs that we strive to meet include (1) a clear value proposition such as time savings; (2) a coherent user and team experience spanning both the digital innovation and the native EHR; (3) a self-explanatory user interface that requires minimal training; and (4) easy access to the innovation within usual workflows. In-depth user needs analyses sometimes uncover unanticipated ways in which the tool can provide additional value, such as by supporting resident education, patient education, or shared decision-making with patients.

### Implementation support should be provided to increase adoption

In addition to user-centered design, adoption of innovations can be increased through implementation support. Approaches we employ include (1) user education through laminated quick reference guides, brief presentations at staff or faculty meetings, and short videos by clinical champions; (2) monitoring and encouraging adoption; (3) tailoring implementation strategies based on the results of user needs assessments; (4) soliciting user feedback through an in-app widget that captures user comments along with a snapshot of the application state; and (5) making every effort to address user feedback in a timely fashion. Another potential approach to increasing adoption is through alerts or non-interruptive reminders recommending use; while potentially effective, this approach should be used with caution due to the potential for alert fatigue.^62^

### Evaluation is critical, but challenging

Evaluation is critical across the project lifecycle. In our experience, however, evaluation is often under-resourced outside of grant-funded initiatives due to never-ending demands for developing new functionality. This problem is compounded by the expertise needed to conduct rigorous evaluations, including for biostatistics, economics, informatics, clinical medicine, and cognitive psychology. We are continuing to refine our roadmap and supportive tooling for conducting such evaluations. In many cases, a rigorous evaluation may require effort comparable to the development of the technology itself, such as when complex clinical decision support logic needs to be replicated for impact assessment. ReImagine EHR has taken some initial steps through the assembly of highly trained evaluation experts and the conduct of several rigorous evaluation studies. However, much more rigorous evaluations are needed to comprehensively assess impact, unintended consequences, and areas for improvement. Ideally, digital innovations should be evaluated through randomized controlled trials conducted across multiple sites and guided by implementation science frameworks.^63^^,^^64^

### App fatigue may be coming

Just as an increasing use of alerts in the EHR led to alert fatigue,^62^ as apps proliferate, it has the potential to lead to “app fatigue.” Potential solutions to such app fatigue include (1) vetting potential additions through formal governance,^65^ (2) delivering a consistent user interface experience aligned with that of the EHR, (3) developing a small number of broad solutions rather than a large number of narrow solutions (eg, one app for managing multiple chronic conditions versus multiple apps for managing individual conditions), (4) embedding links to narrower applications within broader applications, and (5) enhancing or retiring applications with limited usage, user satisfaction, and/or impact.

### Partnerships are essential

Effective execution of a digital innovation initiative such as ReImagine EHR requires interdisciplinary, cross-institutional, and cross-sector partnerships. As demonstrated by the representative innovations described above, successful projects often require contributions from multiple stakeholders. ReImagine EHR relies on interdisciplinary partnerships across diverse domains including clinical medicine, patient engagement, user-centered design, cognitive psychology, standards and interoperability, data science, AI, business, and software engineering. Moreover, since practice patterns and the technology infrastructure may differ significantly across health systems, it is often necessary to engage external institutional partners. Furthermore, industry partners can bring unique resources and expertise. Thus, we believe both internal and external partnerships are essential to maximize impact.

### Opportunities abound

The healthcare community is still in the early stages of applying FHIR-based digital innovations to improve care. There are many exciting, untapped areas for future innovation. Exciting areas for future research and development include direct patient engagement through patient-facing SMART on FHIR applications such as Apple Health^66^; the further application of AI in medicine, such as to predict and proactively provide the information that a provider will likely seek for a given patient; and the integration and use of non-traditional data such as free text narrative in the EHR, sensor data, and payer claims data.

## CONCLUSION

EHR-integrated digital innovation initiatives can be key enterprise assets for improving users’ experience with the EHR, enhancing patient care, and helping address provider burnout. ReImagine EHR provides a case study of how health systems can begin to seize this opportunity.

## FUNDING

This work was supported by the University of Utah, the Agency for Healthcare Research and Quality (R18HS026198 and U18HS027099), the National Cancer Institute of the National Institutes of Health (U24CA204800 and U01CA232826), the National Institute of Diabetes and Digestive and Kidney Diseases of the National Institutes of Health (R18DK123372), the National Center for Advancing Translational Sciences of the National Institutes of Health (3UL1TR002538-03S4), the Office of the National Coordinator for Health Information Technology (90AX0013), the Patient Centered Outcomes Research Institute (PCS-2017C2-7613), the Centers for Medicare & Medicaid Services and the Utah Department of Health (HITECH IAPD 182700537), the Centers for Disease Control and Prevention (NU58DP006770), Hitachi, Ltd., and MD Aware, LLC.

Co-author TJC is supported by a Career Development Award from VA HSR&D (CDA 16-151) and a grant from the VA QUERI National Program (PrOVE QUERI). TR and KLM were supported by the National Library of Medicine of the National Institutes of Health (T15LM007124). Some of the research reported in this publication utilized the Genetic Counseling Shared Resource at Huntsman Cancer Institute at the University of Utah and was supported by the National Cancer Institute of the National Institutes of Health (P30CA042014).

The funding organizations had no role in the conceptualization, design, data collection, analysis, decision to publish, or manuscript preparation for this case study. The content is solely the responsibility of the authors and does not necessarily represent the official views of the organizations involved.

## AUTHOR CONTRIBUTIONS

Each author made substantial contributions to (1) the conception or design of the ReImagine EHR initiative or one of the described projects (all authors); (2) the acquisition, analysis, or interpretation of data (KK, PVK, CW, TJR, HSK, TT, RLC, KM, DB, CHS, JHS, JPH, ST, WT, HB, and GDF); (3) the creation of new software used in the work (KK, PVK, CW, MCF, CN, DKM, PBW, DS, SRL, RLB, and RCC); or (4) the drafting or substantial revision of the manuscript (KK, PVK, CW, TJR, HSK, TT, and GDF). All authors also approved the manuscript for submission and agreed both to be personally accountable for the author’s own contributions and to ensure that questions related to the accuracy or integrity of any part of the work are appropriately investigated, resolved, and documented in the literature.

## References

[1] McDonald CJ. Protocol-based computer reminders, the quality of care and the non-perfectability of man. N Engl J Med 1976; 295 (24): 1351–5. 988482 10.1056/NEJM197612092952405

[2] Institute of Medicine. *To Err Is Human: Building A Safer Health System*. Washington, DC: The National Academies Press; 2000. 25077248

[3] McGlynn EA , Asch SM, Adams J, et al The quality of health care delivered to adults in the United States. N Engl J Med 2003; 348 (26): 2635–45. 12826639 10.1056/NEJMsa022615

[4] Borsky A , Zhan C, Miller T, Ngo-Metzger Q, Bierman AS, Meyers D. Few Americans receive all high-priority, appropriate clinical preventive services. Health Aff (Millwood) 2018; 37 (6): 925–8. 29863918 10.1377/hlthaff.2017.1248

[5] Buntin MB , Jain SH, Blumenthal D. Health information technology: Laying the infrastructure for national health reform. Health Aff 2010; 29 (6): 1214–9. 10.1377/hlthaff.2010.0503 20530358

[6] Stone CP. A glimpse at EHR implementation around the world: the lessons the US can learn. 2014. https://www.e-healthpolicy.org/sites/e-healthpolicy.org/files/A_Glimpse_at_EHR_Implementation_Around_the_World1_ChrisStone.pdf Accessed September 23, 2020.

[7] Cunningham R. Stimulus bill implementation: expanding meaningful use of health IT. Washington (DC): National Health Policy Forum; 2009: 1–16. https://www.nhpf.org/library/issue-briefs/IB834_StimulusIT_08-25-09.pdf Accessed September 23, 2020 19757538

[8] Office of the National Coordinator for Health Information Technology. Percent of hospitals, by type, that possess certified health IT. 2018. https://dashboard.healthit.gov/quickstats/pages/certified-electronic-health-record-technology-in-hospitals.php Accessed September 23, 2020.

[9] Schulte F , Fry E. Death by a thousand clicks: where electronic health records went wrong. Kaiser Health News 2019. https://khn.org/news/death-by-a-thousand-clicks/ Accessed September 23, 2020.

[10] Dastagir MT , Chin HL, McNamara M, Poteraj K, Battaglini S, Alstot L. Advanced proficiency EHR training: effect on physicians’ EHR efficiency, EHR satisfaction and job satisfaction. AMIA Annu Symp Proc 2012; 2012: 136–43. 23304282 PMC3540432

[11] Jamoom EW , Heisey-Grove D, Yang N, Scanlon P. Physician opinions about EHR use by EHR experience and by whether the practice had optimized its EHR use. J Health Med Inform 2016; 7 (4): 1000240. 27800279 10.4172/2157-7420.1000240 PMC5084912

[12] Sinsky C , Colligan L, Li L, et al Allocation of physician time in ambulatory practice: a time and motion study in 4 specialties. Ann Intern Med 2016; 165 (11): 753–60. 27595430 10.7326/M16-0961

[13] Chaiyachati KH , Shea JA, Asch DA, et al Assessment of inpatient time allocation among first-year internal medicine residents using time-motion observations. JAMA Intern Med 2019; 179 (6): 760–7. 30985861 10.1001/jamainternmed.2019.0095 PMC8462976

[14] Hill RG , Sears LM, Melanson SW. 4000 clicks: a productivity analysis of electronic medical records in a community hospital ED. Am J Emerg Med 2013; 31 (11): 1591–4. 24060331 10.1016/j.ajem.2013.06.028

[15] Khairat S , Coleman C, Ottmar P, Jayachander DI, Bice T, Carson SS. Association of electronic health record use with physician fatigue and efficiency. JAMA Netw Open 2020; 3 (6): e207385. 32515799 10.1001/jamanetworkopen.2020.7385 PMC7284310

[16] Melnick ER , Dyrbye LN, Sinsky CA, et al The association between perceived electronic health record usability and professional burnout among US physicians. Mayo Clin Proc 2020; 95 (3): 476–87. 31735343 10.1016/j.mayocp.2019.09.024

[17] Bangor A , Kortum P, Miller J. Determining what individual SUS scores mean: adding an adjective rating scale. J Usability Stud 2009; 4 (3): 114–23.

[18] Kroth PJ , Morioka-Douglas N, Veres S, et al Association of electronic health record design and use factors with clinician stress and burnout. JAMA Netw Open 2019; 2 (8): e199609. 31418810 10.1001/jamanetworkopen.2019.9609 PMC6704736

[19] Levine DM , Linder JA, Landon BE. The quality of outpatient care delivered to adults in the United States, 2002 to 2013. JAMA Intern Med 2016; 176 (12): 1778–90. 27749962 10.1001/jamainternmed.2016.6217

[20] Colicchio TK , Del Fiol G, Scammon DL, Facelli JC, Bowes WA, Narus SP. Comprehensive methodology to monitor longitudinal change patterns during EHR implementations: a case study at a large health care delivery network. J Biomed Inform 2018; 83: 40–53. 29857137 10.1016/j.jbi.2018.05.018

[21] Epic App Orchard—Homepage. https://apporchard.epic.com/ Accessed September 23, 2020.

[22] Cerner App Gallery—Homepage. https://code.cerner.com/apps Accessed September 23, 2020.

[23] Mandel JC , Kreda DA, Mandl KD, Kohane IS, Ramoni RB. SMART on FHIR: A standards-based, interoperable apps platform for electronic health records. J Am Med Inform Assoc 2016; 23 (5): 1–10. 26911829 10.1093/jamia/ocv189 PMC4997036

[24] Unni P , Staes C, Weeks H, et al Why aren’t they happy? An analysis of end-user satisfaction with Electronic health records. AMIA Annu Symp Proc 2016; 2016: 2026–35. 28269962 PMC5333231

[25] Mandl K. SMART on FHIR: big gains, big challenges | Executive Education at HMS. Harvard Medical School. 2019. https://executiveeducation.hms.harvard.edu/industry-insights/smart-fhir-big-gains-big-challenges Accessed September 23, 2020.

[26] ONC’s Cures Act Final Rule. 2020. https://www.healthit.gov/curesrule/ Accessed September 23, 2020.

[27] Kawamoto K , Kukhareva P, Shakib JH, et al Association of an electronic health record add-on app for neonatal bilirubin management with physician efficiency and care quality. JAMA Netw Open 2019; 2 (11): e1915343. 31730181 10.1001/jamanetworkopen.2019.15343 PMC6902796

[28] Boston’s Children’s Hospital. FHIR—Innovation & Digital Health Accelerator. 2020. https://accelerator.childrenshospital.org Accessed September 23, 2020.

[29] Granger BB , Locke SC, Bowers M, et al The digital drag and drop pillbox: design and feasibility of a skill-based education model to improve medication management. J Cardiovasc Nurs 2017; 32 (5): E14–20. 28282304 10.1097/JCN.0000000000000402 PMC5559183

[30] Schleyer TKL , Rahurkar S, Baublet AM, et al Preliminary evaluation of the chest pain dashboard, a FHIR-based approach for integrating health information exchange information directly into the clinical workflow. AMIA Jt Summits Transl Sci Proc 2019; 2019: 656–64. 31259021 PMC6568135

[31] SMART Precision Cancer Medicine. SMART App Gallery. http://apps.smarthealthit.org/app/smart-precision-cancer-medicine Accessed September 23, 2020.

[32] Twichell SA , Rea CJ, Melvin P, et al The effect of an electronic health record–based tool on abnormal pediatric blood pressure recognition. Congenit Heart Dis 2017; 12 (4): 484–90. 28493451 10.1111/chd.12469 PMC5647583

[33] Mandl KD , Gottlieb D, Ellis A. Beyond one-off integrations: a commercial, substitutable, reusable, standards-based, electronic health record–connected app. J Med Internet Res 2019; 21 (2): e12902. 30707097 10.2196/12902 PMC6376332

[34] Open Epic—Homepage. https://open.epic.com/ Accessed September 23, 2020.

[35] Moullin JC , Dickson KS, Stadnick NA, Rabin B, Aarons GA. Systematic review of the Exploration, Preparation, Implementation, Sustainment (EPIS) framework. Implement Sci 2019; 14 (1): 1. 30611302 10.1186/s13012-018-0842-6 PMC6321673

[36] Kushniruk A , Nøhr C. Participatory design, user involvement and health IT evaluation. In: Ammenwerth E Rigby M, eds. Evidence-Based Health Informatics: Promoting Safety and Efficiency through Scientific Methods and Ethical Policy. Vol. 222. Amsterdam, Netherlands: IOS Press; 2016: 139–151. doi:10.3233/978-1-61499-635-4-139. 27198099

[37] Kushniruk AW , Patel VL. Cognitive and usability engineering methods for the evaluation of clinical information systems. J Biomed Inform 2004; 37 (1): 56–76. 15016386 10.1016/j.jbi.2004.01.003

[38] Chokshi SK , Mann DM. Innovating from within: a process model for user-centered digital development in academic medical centers. JMIR Hum Factors 2018; 5 (4): e11048. 30567688 10.2196/11048 PMC6315266

[39] Reese T , Segall N, Nesbitt P, et al Patient information organization in the intensive care setting: Expert knowledge elicitation with card sorting methods. J Am Med Inform Assoc 2018; 25 (8): 1026–35. 30060091 10.1093/jamia/ocy045 PMC6077790

[40] US Core FHIR Profiles. Health Level Seven International (HL7). http://hl7.org/fhir/us/core/ Accessed September 23, 2020.

[41] CDS Hooks Overview. http://cds-hooks.org/ Accessed September 23, 2020.

[42] Kukhareva P , Warner P, Rodriguez S, et al Balancing functionality versus portability for SMART on FHIR applications: case study for a neonatal bilirubin management application. AMIA Annu Symp Proc 2019; 2019: 562–71. 32308850 PMC7153075

[43] SMART App Launch: Scopes and Launch Context. http://www.hl7.org/fhir/smart-app-launch/scopes-and-launch-context/index.html Accessed September 23, 2020.

[44] American Academy of Pediatrics Subcommittee on Hyperbilirubinemia. Management of hyperbilirubinemia in the newborn infant 35 or more weeks of gestation. Pediatrics 2004; 114 (4): 1138. 10.1542/peds.114.1.297 15231951

[45] Provider User Experience App Challenge Awards from the Department of Health and Human Services. Office of the National Coordinator for Health Information Technology. 2017. https://www.challenge.gov/challenge/provider-user-experience-challenge/ Accessed September 23, 2020.

[46] AMIA 2019 Annual Symposium—S74: AMIA/HL7 FHIR^®^ Applications Showcase Session Details. 2019. https://symposium2019.zerista.com/event/member/636489 Accessed September 23, 2020.

[47] Abedin Z , Hoerner R, Habboushe J, et al Implementation of a fast healthcare interoperability resources-based clinical decision support tool for calculating CHA2DS2-VASc scores. Circ Cardiovasc Qual Outcomes 2020; 13 (2): e006286. 32069091 10.1161/CIRCOUTCOMES.119.006286 PMC7266084

[48] Ferng A. Understanding and creating calculators for medical diagnoses. Exclusive Interview with MDCalc. Medgadget 2018; https://www.medgadget.com/2018/06/understanding-and-creating-new-calculators-for-medical-diagnoses-exclusive-interview-with-mdcalc.html Accessed September 23, 2020.

[49] Introduction: standards of medical care in diabetes-2020. Diabetes Care 2020; 43: S1–2. 31862741 10.2337/dc20-Sint

[50] Takeuchi W , Tarumi S, Rodriguez S, et al EHR-integrated, machine learning-driven SMART on FHIR pharmacotherapy decision support system for type-2 diabetes mellitus. In: *AMIA 2018 Informatics Summit*. 2018: 476–7.

[51] Siegel RL , Miller KD, Jemal A. Cancer statistics, 2018. CA Cancer J Clin 2018; 68 (1): 7–30. 29313949 10.3322/caac.21442

[52] Aberle DR , Adams AM, Berg CD, et al Reduced lung-cancer mortality with low-dose computed tomographic screening. N Engl J Med 2011; 365 (5): 395–409. 21714641 10.1056/NEJMoa1102873 PMC4356534

[53] Moyer VA. Screening for lung cancer: U.S. Preventive Services Task Force Recommendation Statement. Ann Intern Med 2014; 160 (5): 330–8. 24378917 10.7326/M13-2771

[54] US Preventive Services Task Force. Final recommendation statement: lung cancer screening. 2016. https://www.uspreventiveservicestaskforce.org/Page/Document/RecommendationStatementFinal/lung-cancer-screening Accessed September 23, 2020.

[55] Richards TB , Soman A, Thomas CC, et al Screening for lung cancer—10 states, 2017. MMWR Morb Mortal Wkly Rep 2020; 69 (8): 201–6. 32106215 10.15585/mmwr.mm6908a1 PMC7367073

[56] Percent Of US. Adults 55 and over with chronic conditions. centers for disease control and prevention. 2020. https://www.cdc.gov/nchs/health_policy/adult_chronic_conditions.htm Accessed September 23, 2020.

[57] Buttorff C , Ruder T, Bauman M. *Multiple Chronic Conditions in the United States*. 2017. https://www.rand.org/pubs/tools/TL221.html Accessed September 23, 2020.

[58] National Health Expenditures 2017 Highlights. Center for Medicare & Medicaid Services. https://www.cms.gov/Research-Statistics-Data-and-Systems/Statistics-Trends-and-Reports/NationalHealthExpendData/Downloads/highlights.pdf Accessed September 23, 2020.

[59] Curran RL , Kukhareva PV, Taft T, et al Integrated displays to improve chronic disease management in ambulatory care: A SMART on FHIR application informed by mixed-methods user testing. J Am Med Inform Assoc 2020; 27 (8): 1225–34. 32719880 10.1093/jamia/ocaa099 PMC7481023

[60] Conrad DA , Grembowski D, Hernandez SE, Lau B, Marcus-Smith M. Emerging lessons from regional and state innovation in value-based payment reform: balancing collaboration and disruptive innovation. Milbank Q 2014; 92 (3): 568–623. 25199900 10.1111/1468-0009.12078 PMC4221757

[61] COVID-19 Toolkit—App Orchard. 2020. https://apporchard.epic.com/Gallery/Index?id=5692 Accessed September 23, 2020.

[62] McCoy AB , Thomas EJ, Krousel-Wood M, Sittig DF. Clinical decision support alert appropriateness: a review and proposal for improvement. Ochsner J 2014; 14 (2): 195–202. 24940129 PMC4052586

[63] Haynes RB , del Fiol G, Michelson M, Iorio A. Context and approach in reporting evaluations of electronic health record-based implementation projects. Ann Intern Med 2020; 172(11_Supplement): S73–8. 32479174 10.7326/M19-0874

[64] Kawamoto K , McDonald CJ. Designing, conducting, and reporting clinical decision support studies: recommendations and call to action. Ann Intern Med 2020; 172 (11): S101–9. 32479177 10.7326/M19-0875

[65] Kawamoto K , Flynn MC, Kukhareva P, et al A pragmatic guide to establishing clinical decision support governance and addressing decision support fatigue: a case study. AMIA Annu Symp Proc 2018; 2018: 624–33. 30815104 PMC6371304

[66] Apple Health. https://www.apple.com/healthcare/health-records/ Accessed September 23, 2020.

